# Drift in Depression Prevalence Disorder in Gulf Cooperation Council (GCC) Countries Over 30 Years

**DOI:** 10.1192/bjo.2024.175

**Published:** 2024-08-01

**Authors:** Mitha Al Balushi, Fatima Al Maskari, Syed Javaid, Amar Ahamd

**Affiliations:** 1 Public Health Research Center, New York University-Abu Dhabi, Abu Dhabi, UAE; 2 Institute of Public Health, College of Medicine and Health Sciences, Abu Dhabi, UAE; 3 Department of Psychiatry and Behavioral Sciences, College of Medicine and Health Sciences, Abu Dhabi, UAE

## Abstract

**Aims:**

Depression disorder is a major public health problem and a serious medical illness which negatively affects people's daily life. The WHO's International Classification of Diseases (ICD–10) defines this set of disorders ranging from mild to moderate to severe. Estimated annual percentage change (EAPC) is a useful statistic that is used to measure trends in rates over time-period.

The aim of this study was to compute the drift in depression prevalence disorder using the EAPC of the prevalence of depression disorder between 1990 to 2019 with corresponding 95% confidence intervals (95% CI) across the GCC countries.

**Methods:**

Prevalence of depression disorder data for the GCC countries were downloaded from “Our World in Data” https://ourworldindata.org/mental-health#depression. We computed the drift of depression over 30 years between the 6 GCC countries using the statistical software R.

**Results:**

The greatest decrease was seen for Bahrain which is (–5.2%) followed by Qatar (–3.2%) and United Arab Emirates (–3%). However, the largest increase was observed for Saudi Arabia (2.7%), followed by Kuwait (1.1%) and Oman (0.7%). The reduction in the prevalence of depression disorder seen in Bahrain, Qatar and United Arab Emirates shows a significant achievement in mental health diagnosis, prevention, and treatment.

**Conclusion:**

However, further studies are required to better understand the drifts in the GCC countries. Furthermore, governmental funding for academic and research mental health programs is highly recommended.

